# Risk Factors and Long‐Term Prognosis for Coinfection of Nontuberculous Mycobacterial Pulmonary Disease and Chronic Pulmonary Aspergillosis: A Multicentre Observational Study in Japan

**DOI:** 10.1111/myc.70083

**Published:** 2025-06-19

**Authors:** Yasuhiro Tanaka, Shotaro Ide, Takahiro Takazono, Kazuaki Takeda, Naoki Iwanaga, Masataka Yoshida, Naoki Hosogaya, Yusei Tsukamoto, Satoshi Irifune, Takayuki Suyama, Tomo Mihara, Akira Kondo, Tsutomu Kobayashi, Yuichi Fukuda, Eisuke Sasaki, Toyomitsu Sawai, Yasuhito Higashiyama, Kohji Hashiguchi, Minako Hanaka, Toshihiko Ii, Kiyoyasu Fukushima, Kosaku Komiya, Taiga Miyazaki, Kazuhiro Yatera, Koichi Izumikawa, Akitsugu Furumoto, Katsunori Yanagihara, Hiroshi Mukae

**Affiliations:** ^1^ Department of Respiratory Medicine Nagasaki University Graduate School of Biomedical Sciences Nagasaki Japan; ^2^ Department of Respiratory Medicine Sasebo City General Hospital Sasebo Japan; ^3^ Infectious Diseases Experts Training Center Nagasaki University Hospital Nagasaki Japan; ^4^ Department of Respiratory Medicine Nagasaki University Hospital Nagasaki Japan; ^5^ Department of Infectious Diseases Nagasaki University Graduate School of Biomedical Sciences Nagasaki Japan; ^6^ Clinical Research Center Nagasaki University Hospital Nagasaki Japan; ^7^ Department of Internal Medicine Izumikawa Hospital Nagasaki Japan; ^8^ Department of Internal Medicine Nagasaki Prefecture Shimabara Hospital Shimabara Japan; ^9^ Department of Respiratory Medicine Nagasaki Gotochuoh Hospital Goto Japan; ^10^ Department of Respiratory Medicine Japan Community Health Care Organization Isahaya General Hospital Isahaya Japan; ^11^ Department of Respiratory Medicine NHO Nagasaki Medical Center Omura Japan; ^12^ Department of Respiratory Medicine Sasebo Chuo Hospital Sasebo Japan; ^13^ Department of Respiratory Medicine NHO Ureshino Medical Center Ureshino Japan; ^14^ Department of Respiratory Medicine Nagasaki Harbor Medical Center Nagasaki Japan; ^15^ Department of Internal Medicine Hokusho Central Hospital Sasebo Japan; ^16^ Department of Respiratory Medicine Japanese Red Cross Nagasaki Genbaku Hospital Nagasaki Japan; ^17^ Respiratory Medicine Iizuka Hospital Iizuka Japan; ^18^ Department of Respiratory Medicine NHO Miyazaki Higashi Hospital Miyazaki Japan; ^19^ Department of Respiratory Medicine Japanese Red Cross Nagasaki Genbaku Isahaya Hospital Isahaya Japan; ^20^ Respiratory Medicine and Infectious Diseases Oita University Faculty of Medicine Yufu Japan; ^21^ Research Center for Global and Local Infectious Diseases Oita University Yufu Japan; ^22^ Division of Respirology, Rheumatology, Infectious Diseases and Neurology, Department of Internal Medicine University of Miyazaki Miyazaki Japan; ^23^ Department of Respiratory Medicine University of Occupational and Environmental Health, Japan Kitakyushu Japan; ^24^ Department of Laboratory Medicine Nagasaki University Hospital Nagasaki Japan

**Keywords:** *Aspergillus*, coinfection, *Mycobacterium avium* complex, *Mycobacterium* infections, nontuberculous mycobacteria, propensity score, pulmonary aspergillosis, retrospective studies

## Abstract

**Background:**

Nontuberculous mycobacterial pulmonary disease (NTM‐PD) is a chronic respiratory infection with increasing prevalence and mortality worldwide. Coinfection with chronic pulmonary aspergillosis (CPA) is a significant complication of NTM‐PD, often complicating treatment and resulting in poor prognosis.

**Objective:**

In this multicentre, retrospective cohort study, we examined the epidemiology, comorbidities, risk factors for CPA coinfection and long‐term prognosis of patients with NTM‐PD infected with CPA in Japan.

**Methods:**

Patients aged ≥ 18 years with newly diagnosed NTM‐PD who visited 18 hospitals between 2010 and 2017 in Kyushu, Japan, were included. Medical records were reviewed for patient characteristics, mycobacterial species, laboratory data, radiological features, *Aspergillus* coinfection and all‐cause mortality rates. Risk factors for CPA coinfection were analysed using multiple logistic regression, and survival analysis was performed before and after propensity score matching with risk factors.

**Results:**

Among 1304 patients with NTM‐PD, 45 (3.5%) were diagnosed with CPA, including 42 with chronic progressive pulmonary aspergillosis. The risk factors for CPA coinfection included male sex, chronic obstructive pulmonary disease, oral corticosteroid use and cavity formation. All‐cause mortality was significantly higher in patients with NTM‐PD with CPA than in those without CPA (log‐rank test, *p* < 0.001; crude hazard ratio [HR], 3.98). Survival analysis after propensity score matching suggested CPA was an independent poor prognostic factor (log‐rank test, *p* = 0.036; adjusted HR, 1.59).

**Conclusion:**

CPA is an independent poor prognostic factor in patients with NTM‐PD. Clinicians must consider CPA when treating patients with NTM‐PD, particularly those with high‐risk factors, to ensure timely diagnosis and management.

## 1 Introduction

Nontuberculous mycobacteria (NTM) are ubiquitous environmental microorganisms that cause various human infections. Nontuberculous mycobacterial pulmonary disease (NTM‐PD) is a chronic respiratory infection caused by NTM and has been recognised as an important respiratory infection in recent years. Recent reports have indicated a worldwide increase in NTM‐PD prevalence, with Japan showing particularly high rates and increasing mortality [1, 2, 3, 4]. *Mycobacterium avium* and *Mycobacterium intracellulare* are the most common NTM species, accounting for 90% of NTM‐PD cases in Japan [2, 5]. NTM‐PD requires a long‐term multidrug treatment regimen; however, it is sometimes refractory and recurs after treatment [6].

Recently, chronic pulmonary aspergillosis (CPA) has been identified as an important complication of NTM‐PD, which typically leads to a poor prognosis [7, 8, 9]. While both NTM‐PD and CPA individually require long‐term treatment, coinfection makes treatment difficult because of the drug–drug interactions between rifamycins, macrolides and azole antifungal agents [10]. Moreover, CPA is associated with the exacerbation of chronic infections and increased mortality rates in patients with NTM‐PD. Previous studies have elucidated the risk factors, comorbidity rates and prognostic implications of CPA in patients with NTM‐PD [7, 8, 9, 11]. However, most of this knowledge stems from investigations conducted at single centres or studies with constrained sample sizes. Therefore, we conducted a multicentre, retrospective cohort study of patients with NTM‐PD in Kyushu, Japan, including tertiary care and community hospitals. We aimed to determine the epidemiology, comorbidities, risk factors and long‐term prognosis of patients with NTM‐PD coinfected with CPA.

## 2 Methods

### 2.1 Study Design

This multicentre, retrospective, observational cohort study was conducted at 18 acute care hospitals in Kyushu, Japan (Table S1): Nagasaki, Fukuoka, Oita, Miyazaki and Saga (NFOMS NTM study). Patients aged ≥ 18 years with newly diagnosed NTM‐PD who visited the study centres between January 1, 2010, and December 31, 2017, were included. The exclusion criteria were death before a definitive diagnosis of NTM‐PD, referral to another institution for initial treatment after diagnosis, and last observation conducted before the date of confirmed diagnosis. Medical records were reviewed by respiratory medicine specialists at each facility for patient characteristics, underlying diseases, isolated mycobacterial species, laboratory data and radiological features when the patient was definitively diagnosed with NTM‐PD, chronic infections, treatments and outcomes during the study period. Patients were followed up until December 31, 2022. The primary endpoint of this study was to determine whether CPA coinfection was an independent poor prognostic factor for NTM‐PD. The secondary endpoint was the assessment of CPA coinfection rates and associated risk factors. All data were collected using the Research Electronic Data Capture (REDCap) hosted at Nagasaki University Hospital. The authors confirm that the ethical policies of the journal, as noted on the journal's author guidelines page, have been adhered to, and the appropriate ethical review committee approval has been received. This study was conducted in accordance with the guidelines of the Declaration of Helsinki and approved by the Institutional Review Board of Nagasaki University Hospital (approval number: 22121903). Verbal informed consent was obtained from all participants whenever possible. As this was a retrospective observational study, an opt‐out procedure was provided to the participants.

### 2.2 Definitions for NTM‐PD

The diagnostic criteria established in 2008 by the Japanese Society for Tuberculosis and Nontuberculous Mycobacteriosis (JSTNM) were used to diagnose NTM‐PD [12]. These criteria are congruent with those of the American Thoracic Society/European Respiratory Society/European Society of Clinical Microbiology and Infectious Diseases/Infectious Diseases Society of America Clinical Practice Guidelines 2020. However, the JSTNM diagnostic criteria do not include clinical symptoms [6, 13]. NTM species identification methods vary according to the institution and include PCR for *M. avium* and *M. intracellulare* , DNA–DNA hybridisation, and matrix‐assisted laser desorption/ionisation‐time‐of‐flight mass spectrometry. The radiological classification was based on the first computed tomography of the chest at the NTM‐PD diagnosis and classified by experts as either nodular‐bronchiectatic (NB) pattern, NB with cavity pattern, fibrocavitary pattern, single nodule, hypersensitivity pneumonitis or others.

### 2.3 CPA Diagnosis

CPA was diagnosed based on the criteria of the Japanese Domestic Guidelines for Management of Deep‐seated Mycosis 2014 [14]. Briefly, these criteria include clinical symptoms, radiological findings, serological tests, including *Aspergillus* precipitating antibody, serum *Aspergillus* galactomannan antigen, and β‐1,3‐D‐glucan, and proof of *Aspergillus* species using pathology or culture. *Aspergillus* precipitating antibody, which predominantly reflects Immunoglobulin G (IgG) antibody, was detected by FSK1 *Aspergillus* immunodiffusion system (Microgen Bioproducts Ltd., UK) in Nagasaki University Hospital and outsourcing laboratory testing in other hospitals. These diagnostic criteria included CPA, such as simple aspergilloma, chronic cavitary pulmonary aspergillosis (CCPA), chronic fibrosing pulmonary aspergillosis (CFPA), *Aspergillus* nodule and subacute invasive pulmonary aspergillosis (SAIA), according to the European Respiratory Society/European Society of Clinical Microbiology and Infectious Diseases guidelines [15]. According to the Japanese guidelines, CCPA, CFPA and SAIA were comprehensively included as chronic progressive pulmonary aspergillosis. Patients with *Aspergillus* spp. colonisation were excluded from the CPA group.

### 2.4 Statistical Analysis

Risk factors for NTM‐PD with CPA were analysed using the Mann–Whitney *U* test for continuous variables and the Fisher exact test for nominal variables. Multiple logistic regression analysis was performed by selecting items that were deemed clinically important by the experts. Risk factors with a frequency of < 5 were excluded from the analyses. Propensity score matching was performed between patients with NTM‐PD without CPA and those with NTM‐PD with CPA using the results obtained from the multiple logistic regression analysis. Nearest‐neighbour matching at a ratio of 1:5 was performed using a 0.2 calliper. A standardised difference of < 0.10 indicated a sufficient balance between the groups. Survival analysis was performed for the NTM‐PD groups with and without CPA using the log‐rank test and Cox proportional hazards model before and after propensity score matching. All *p*‐values were two‐sided, and *p*‐values ≤ 0.05 were considered to be statistically significant. All statistical analyses were performed using EZR (version 1.67; Saitama Medical Center, Jichi Medical University, Saitama, Japan), a graphical user interface for R (version 4.3.3; The R Foundation for Statistical Computing, Vienna, Austria) [16]. It is a modified version of the R commander, designed to add statistical functions frequently used in biostatistics.

## 3 Results

### 3.1 Patient Characteristics

**FIGURE 1:**
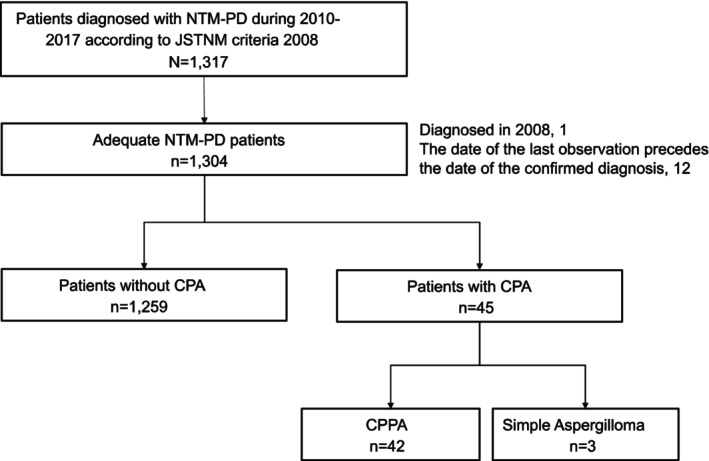
Flowchart of study patients. During the study period, 1317 patients were newly diagnosed with NTM‐PD, and 1304 were evaluated. The median observation period was 59 months (IQR: 19–95 months). Of these, 45 patients (3.5%) were diagnosed with CPA, and there were three cases of simple aspergilloma and 42 cases of CPPA, including CCPA, CFPA, and SAIA. CCPA, chronic cavitary pulmonary aspergillosis; CFPA, chronic fibrosing pulmonary aspergillosis; CPA, chronic pulmonary aspergillosis; CPPA, chronic progressive pulmonary aspergillosis; IQR, interquartile range; JSTNM, The Japanese Society for Tuberculosis and Nontuberculous Mycobacteriosis; NTM‐PD, nontuberculous mycobacterial pulmonary disease; SAIA, subacute invasive pulmonary aspergillosis.

During the study period, 1317 patients were newly diagnosed with NTM‐PD, and 1304 patients were evaluated (Figure 1). The median observation period was 59 months (interquartile range [IQR]: 19–95 months). Patient characteristics are shown in Table 1. Of these, 45 patients (3.5%) were diagnosed with CPA during the observation period: 12 were diagnosed with CPA before NTM‐PD diagnosis, 13 were diagnosed with CPA concurrently with NTM‐PD, and 20 were diagnosed with CPA after NTM‐PD diagnosis. In the CPA subtype, there were three patients with simple aspergilloma and 42 patients with chronic progressive pulmonary aspergillosis, including CCPA, CFPA and SAIA.

**TABLE 1:** Characteristics of patients with nontuberculous mycobacterial pulmonary disease at diagnosis.

		NTM‐PD without CPA	NTM‐PD with CPA
	*N* = 1304 (%)	*n* = 1259 (%)	*n* = 45 (%)
Age (years), mean (SD)	70.7 ± 11.5	70.8 ± 11.6	68.9 ± 10.0
Sex, male	423 (32.4)	393 (31.2)	30 (66.7)
Body mass index (kg/cm^2^) (*n* = 1090), mean (SD)	19.6 ± 3.1	19.7 ± 3.0	18.4 ± 3.3
Smoking habit	310 (23.8)	286 (22.7)	24 (53.3)
Pulmonary comorbidities	353 (27.1)	325 (25.8)	28 (53.3)
Old tuberculosis	100 (7.7)	91 (7.2)	9 (20.0)
Bronchiectasis	99 (7.6)	90 (7.1)	9 (20.0)
Interstitial lung disease	67 (5.1)	57 (4.5)	10 (22.2)
Lung tumour	49 (3.8)	48 (3.8)	1 (2.2)
COPD	45 (3.5)	34 (2.7)	11 (24.4)
Pulmonary emphysema	43 (3.3)	34 (2.7)	9 (20.0)
Bronchial asthma	38 (2.9)	36 (2.9)	2 (4.4)
Chronic bronchitis	33 (2.5)	29 (2.3)	4 (8.9)
Post lung operation	25 (1.9)	22 (1.7)	3 (6.7)
Pneumoconiosis	16 (1.2)	14 (1.1)	2 (4.4)
Prior Chronic pulmonary aspergillosis	13 (1.0)	2 (0.2)	11 (24.4)
Chronic respiratory failure with home oxygen therapy	4 (0.3)	3 (0.2)	1 (2.2)
Asbestos‐related pleural disease	4 (0.3)	3 (0.2)	1 (2.2)
Pulmonary sarcoidosis	1 (0.1)	1 (0.1)	0 (0.0)
Systemic comorbidities			
Solid tumour, localised in recent 5‐years	160 (12.3)	156 (12.4)	4 (8.9)
Connective tissue diseases	113 (8.7)	105 (8.3)	8 (17.8)
Diabetes mellitus	97 (7.4)	93 (7.4)	4 (8.9)
Cerebrovascular disease	47 (3.6)	44 (3.5)	3 (6.7)
Liver disease, moderate/severe	43 (3.3)	42 (3.3)	1 (2.2)
Congestive heart failure	33 (2.5)	31 (2.5)	2 (4.4)
Solid tumour, metastatic in recent 5‐years	33 (2.5)	32 (2.5)	1 (2.2)
Myocardial infarction	31 (2.4)	30 (2.4)	1 (2.2)
Dementia	29 (2.2)	29 (2.3)	0 (0.0)
Lymphoma, active or in remission	24 (1.8)	23 (1.8)	1 (2.2)
Peptic ulcer disease	19 (1.5)	17 (1.4)	2 (4.4)
Renal disease	18 (1.4)	16 (1.3)	2 (4.4)
Peripheral vascular disease	7 (0.5)	7 (0.6)	0 (0.0)
Leukaemia, active or in remission	5 (0.4)	5 (0.4)	0 (0.0)
AIDS, without asymptomatic HIV infection	3 (0.2)	3 (0.2)	0 (0.0)
Paralysis	2 (0.2)	2 (0.2)	0 (0.0)
Medications for comorbidities	166 (12.7)	151 (12.0)	15 (33.3)
Oral corticosteroids	98 (7.5)	86 (6.8)	12 (26.7)
Inhaled corticosteroids	27 (2.1)	25 (2.0)	2 (4.4)
Immunosuppressants	67 (5.1)	59 (4.7)	8 (17.8)
Chemotherapy	18 (1.4)	17 (1.4)	1 (2.2)
Biological agents	8 (0.6)	7 (0.6)	1 (2.2)
Laboratory Data, mean (SD)			
Albumin (g/dL) (*n* = 1111)	3.8 ± 0.7	3.8 ± 0.7	3.4 ± 0.5
ESR (mm/h) (*n* = 532)	34 ± 26	33.2 ± 25.8	57.0 ± 33.3
White blood cell count (/μL) (*n* = 1226)	6275 ± 2773	6205 ± 2739	8207 ± 3024
Lymphocyte count (/μL) (*n* = 1215)	1429 ± 704	1424 ± 650	1575 ± 1595
Serum galactomannan antigen (COI) (*n* = 303)	0.72 ± 0.93	0.71 ± 0.91	0.88 ± 1.13
Radiological features (*n* = 1200)			
Bronchiectasis	990 (75.9)	951 (75.5)	39 (86.7)
Cavity formation	343 (26.3)	309 (24.5)	34 (75.6)
Cavity formation ≥ 2 cm	192 (14.7)	169 (13.4)	23 (51.1)
Cavity formation ≥ 2 lobes	112 (8.6)	98 (7.8)	14 (31.1)
Fungus ball	19 (1.5)	10 (0.8)	9 (20.0)
Radiological patterns (*n* = 1200)			
Non‐cavitary NB pattern	785 (60.2)	779 (61.9)	6 (13.3)
Cavitary NB pattern	241 (18.5)	222 (17.6)	19 (42.2)
Fibrocavitary pattern	60 (4.6)	49 (3.9)	11 (24.4)
Single nodule	58 (4.4)	57 (4.5)	1 (2.2)
Hypersensitivity pneumonitis	2 (0.2)	2 (0.2)	0 (0.0)
Others	54 (4.1)	50 (4.0)	4 (8.9)
Observation period (month), mean (SD)	60.9 ± 44.6	61.2 ± 44.6	53.8 ± 43.0

Abbreviations: AIDS, acquired immunodeficiency syndrome; COI, cutoff index; COPD, chronic obstructive pulmonary disease; CPA, chronic pulmonary aspergillosis; ESR, erythrocyte sedimentation rate; NB, nodular bronchiectasis; NTM‐PD, nontuberculous mycobacterial pulmonary disease; SD, standard deviation.

**FIGURE 2:**
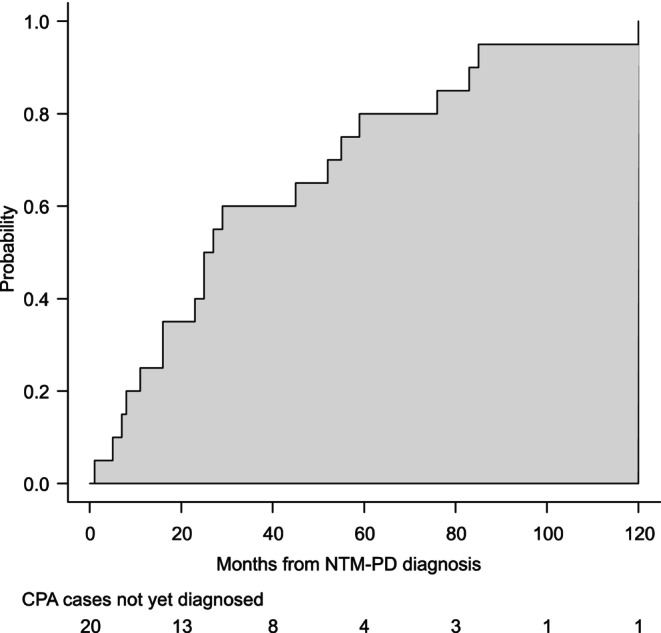
Duration from nontuberculous mycobacterial pulmonary disease diagnosis to chronic pulmonary aspergillosis diagnosis. The median duration from NTM‐PD diagnosis to CPA diagnosis was 26 months (IQR, 12–58 months), with a mean of 38.4 ± 32.6 months. CPA, chronic pulmonary aspergillosis; IQR, interquartile range; NTM‐PD, nontuberculous mycobacterial pulmonary disease.

The median time from NTM‐PD diagnosis to CPA diagnosis was 26 months (IQR 12–58 months) (Figure 2). Among patients with NTM‐PD presenting with CPA, cavitary lesions were common in 34 (75.6%), with 9 patients (20%) having a fungus ball and 1 (2.2%) having a single nodule. Of the 45 patients with NTM‐PD infected with CPA, 35 were treated for CPA during the observation period (9 before and 26 after diagnosis of NTM‐PD), and 5 patients required a change in NTM‐PD medication for CPA treatment. The initial antifungal agents were voriconazole in 13 patients (28.9%) and itraconazole and micafungin in 6 patients each (13.3%) (Table S2). Surgical resection for CPA was performed in 4 (8.9%) patients.

### 3.2 Isolated *Mycobacterial* and *Aspergillus* Species

In this study, 1328 *Mycobacterium* strains were isolated and identified in 1304 patients (Table S3). *Mycobacterium intracellulare* was the most common cause of NTM‐PD (50.4%), followed by *M. avium* (40.5%), *M. abscessus* (2.4%), *M. kansasii* (2.4%) and *M. avium‐intracellulare* complex (1.7%) infections. Of the 45 patients diagnosed with CPA, 24 *Aspergillus* strains were isolated in 21 patients: *Aspergillus fumigatus* in 14 (66.7%), *A. niger* in 4 (19.0%), *A. terreus* in 3 (14.3%) and *A. flavus* , *A. nidulans* , and unidentified in one patient each (4.8%). Of these, two patients had *A. fumigatus* and *A. terreus* coinfection, and one had *A. fumigatus* and *A. niger* coinfection. *Aspergillus* antibody was positive in 12/14 cases, and non‐fumigatus *Aspergillus* single infections were all negative. Six patients with *Aspergillus* colonisation ( *A. fumigatus* , *n* = 2; *A. niger* , *n* = 1; *A. versicolor* , *n* = 1; and unidentified, *n* = 2) were excluded from the CPA group.

### 3.3 Risk Factors for Aspergillus Coinfection

To examine the risk factors for NTM‐PD with CPA coinfection, we compared the patient background at the time of new diagnosis of NTM‐PD in 1259 patients with NTM‐PD without CPA and 45 patients with NTM‐PD with CPA, out of a total of 1304 patients with confirmed NTM‐PD. Univariate analysis revealed that male sex, low body mass index, smoking history, old tuberculosis, bronchiectasis, interstitial lung disease, chronic obstructive pulmonary disease (COPD), pulmonary emphysema, chronic bronchitis, pulmonary aspergillosis, any dose of oral corticosteroids and immunosuppressants, hypoalbuminemia, elevation of erythrocyte sedimentation rate, cavity formation and radiological cavitary and fibrocavitary NB patterns were risk factors for CPA complications. Multiple logistic regression analysis was performed using age, male sex, COPD, oral corticosteroid use and cavity formation. This analysis showed that male sex, COPD, oral corticosteroid use and cavity formation were risk factors for CPA (Table 2 and Table S4).

**TABLE 2:** Characteristics of patients with NTM‐PD with and without CPA.

	NTM‐PD without CPA	NTM‐PD with CPA	Univariate analysis		Multivariate analysis	
	*n*	*n*	Crude OR (95% CI)	*p*	Adjusted OR (95% CI)	*p*
	1259 (%)	45 (%)				
Age (years), mean (SD)	70.8 ± 11.6	68.9 ± 10.0		0.162	0.99 (0.97, 1.02)	0.487
Sex, male	393 (31.2)	30 (66.7)	4.40 (2.26, 8.91)	< 0.001	2.34 (1.17, 4.70)	0.016
Body mass index (kg/cm^2^) (*n* = 1090), mean (SD)	19.7 ± 3.0	18.4 ± 3.3		0.005		
Smoking habit (*n* = 994)	286 (22.7)	24 (53.3)	3.87 (1.98, 7.69)	< 0.001		
Pulmonary comorbidities						
Old tuberculosis	91 (7.2)	9 (20.0)	3.20 (1.32, 7.05)	0.006		
Bronchiectasis	90 (7.1)	9 (20.0)	3.24 (1.33, 7.14)	0.005		
Interstitial lung disease	57 (4.5)	10 (22.2)	6.01 (2.52, 13.18)	< 0.001		
Lung tumour	48 (3.8)	1 (2.2)	0.57 (0.01, 3.53)	1.000		
COPD	34 (2.7)	11 (24.4)	11.60 (4.88, 25.97)	< 0.001	4.44 (1.86, 10.60)	< 0.001
Pulmonary emphysema	34 (2.7)	9 (20.0)	8.97 (3.52, 20.95)	< 0.001		
Bronchial asthma	36 (2.9)	2 (4.4)	1.58 (0.18, 6.49)	0.381		
Chronic bronchitis	29 (2.3)	4 (8.9)	4.13 (1.01, 12.57)	0.024		
Post lung operation	22 (1.7)	3 (6.7)	4.01 (0.74, 14.15)	0.052		
Pneumoconiosis	14 (1.1)	2 (4.4)	4.13 (0.44, 18.87)	0.103		
Medications						
Oral corticosteroids	86 (6.8)	12 (26.7)	4.95 (2.24, 10.27)	< 0.001	4.76 (2.19, 10.30)	< 0.001
Inhaled corticosteroids	25 (2.0)	2 (4.4)	2.29 (0.26, 9.71)	0.238		
Immunosuppressants	59 (4.7)	8 (17.8)	4.39 (1.69, 10.14)	0.002		
Laboratory data, mean (SD)						
Albumin (g/dL) (*n* = 1111)	3.8 ± 0.7	3.4 ± 0.5		< 0.001		
ESR (mm/h) (*n* = 532)	33.2 ± 25.8	57.0 ± 33.3		< 0.001		
White blood cell count (/μL) (*n* = 1226)	6205 ± 2739	8207 ± 3024		< 0.001		
Lymphocyte count (/μL) (*n* = 1215)	1424 ± 650	1575 ± 1595		0.414		
Radiological features (*n* = 1200)						
Bronchiectasis	951 (75.5)	39 (86.7)	2.10 (0.87, 6.12)	0.109		
Cavity formation	309 (24.5)	34 (75.6)	9.47 (4.62, 20.99)	< 0.001	7.1 (3.45, 14.60)	< 0.001
Cavity formation ≥ 2 cm	169 (13.4)	23 (51.1)	6.72 (3.49, 12.96)	< 0.001		
Cavity formation ≥ 2 lobes	98 (7.8)	14 (31.1)	1.66 (0.71, 3.87)	0.226		
Fungus ball	10 (0.8)	9 (20.0)	30.79 (10.40, 90.32)	< 0.001		
Radiological patterns (*n* = 1200)						
Non‐cavitary NB pattern	779 (61.9)	6 (13.3)	Reference			
Cavitary NB pattern	222 (17.6)	19 (42.2)	11.08 (4.19, 34.34)	< 0.001		
Fibrocavitary pattern	49 (3.9)	11 (24.4)	28.81 (9.31, 99.13)	< 0.001		
All‐cause mortality at 5 years	14.9%	42.8%		< 0.001^a^		
All‐cause mortality at 10 years	24.3%	75.7%		< 0.001^a^		

Abbreviations: CI, confidence interval; COPD, chronic obstructive pulmonary disease; CPA, chronic pulmonary aspergillosis; ESR, erythrocyte sedimentation rate; NB, nodular bronchiectasis; NTM‐PD, nontuberculous mycobacterial pulmonary disease; OR, odds ratio; SD, standard deviation.

^a^ Log‐rank test.

### 3.4 Survival Analysis for All‐Cause Mortality

**FIGURE 3:**
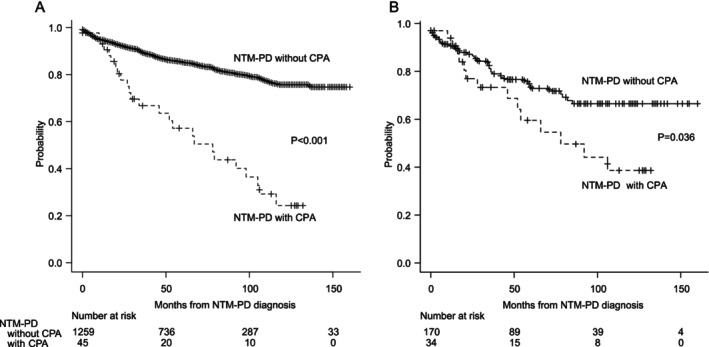
Kaplan–Meier analysis for patients with NTM‐PD with or without CPA, before (A) and after (B) propensity score matching. Patients with NTM‐PD infected with CPA had significantly worse prognoses than those without CPA (A). Analysis after propensity score matching for age, male sex, COPD, oral corticosteroid use, and cavity formation showed that CPA was a significantly poor prognostic factor (B). COPD, chronic obstructive pulmonary disease; CPA, chronic pulmonary aspergillosis; NTM‐PD, nontuberculous mycobacterial pulmonary disease.

Among patients with NTM‐PD without CPA (*n* = 1259) and with CPA (*n* = 45), the all‐cause mortality from the diagnosis of NTM‐PD to the date of the last observation was 15.3% (*n* = 192) and 55.6% (*n* = 25), respectively (log‐rank test, *p* < 0.001; crude HR, 3.98; 95% confidence interval [CI], 2.62–6.05). Patients with CPA had a significantly worse prognosis than those without CPA (Table 3). Kaplan–Meier curves are shown in Figure 3A. To determine whether the presence of CPA was an independent poor prognostic factor or whether the patient's predisposition to CPA was a poor prognostic factor, we performed propensity score matching for age, male sex, COPD, oral corticosteroid use and cavity formation. The five values used for matching had a standardised mean difference of < 0.10 (Table S5). After matching, the CPA group continued to show a significantly poor prognosis according to the Kaplan–Meier curves (log‐rank test, *p* = 0.036) (Figure 3B). However, in the Cox proportional hazards model, the adjusted HR was 1.59 (95% CI 0.84–3.02), which did not reach statistical significance (Table 3).

**TABLE 3:** Comparison of all‐cause mortality.

	Before propensity score matching		After propensity score matching	
	Number of events/All cases	Crude HR (95% CI)	Number of events/All cases	Adjusted HR (95% CI)
NTM‐PD without CPA	192/1259	Reference	43/170	Reference
NTM‐PD with CPA	25/45	3.98 (2.62–6.05)	15/34	1.59 (0.84–3.02)

Abbreviations: CI, confidence interval; CPA, chronic pulmonary aspergillosis; HR, hazards ratio; NTM‐PD, nontuberculous mycobacterial pulmonary disease.

## 4 Discussion

The results of this multicentre study provide significant insights into CPA coinfection in patients with NTM‐PD. Our key findings include the CPA coinfection rate, identification of specific risk factors, and demonstration of its impact on long‐term prognosis.

The observed CPA coinfection rate in our cohort was lower than that reported in previous observational studies (3.5% vs. 3.9%–11.0%) [7, 8, 9, 11, 17, 18, 19]. This discrepancy may be attributed to the heterogeneity of medical facilities and variability in CPA diagnostic practices. Moreover, the aforementioned studies were conducted at single‐centre hospitals, potentially resulting in a substantial number of patients with NTM‐PD with complex disease states owing to selection bias. Sehgal et al. reported the prevalence of CPA among patients with post‐tuberculous or non‐tuberculous mycobacterial lung infections through a systematic review, and the coinfection rate was 7% among patients with NTM‐PD. They reported heterogeneity, publication bias, and overestimation in hospital settings [19]. Our study included 1304 patients from 18 medical facilities, including non‐tertiary care centres, potentially encompassing representative patients with NTM‐PD, including those with mild infection. Furthermore, CPA diagnosis is challenging for various reasons. The detection rate of fungal microscopy and fungal culture of respiratory specimens is low; β‐1,3‐D‐glucan is not specific for *Aspergillus* in serological tests, galactomannan antigen is not sensitive, and anti‐*Aspergillus* precipitating antibodies are specific but not sensitive, particularly for non‐fumigatus species [20]. This implies that the diagnostic accuracy of CPA varies among facilities. However, the impact is likely to be small because all facilities participating in this study were able to test for bronchoscopy, β‐1,3‐D‐glucan, galactomannan antigen, and antibody. We have previously reported on NTM‐PD and CPA complications based on the Japanese large‐scale claims database [21]. The reported incidence of CPA in NTM‐PD was 2.29%, which is lower than that reported in the current study. However, the previous report included patients treated for both NTM‐PD and CPA, whereas the coinfection rate in the current report included all patients, regardless of treatment, and thus better reflects the real‐world coinfection rate of NTM‐PD and CPA.

In this study, *M. intracellulare* and *M. avium* were dominant. *A. fumigatus* was the most common species causing CPA, followed by *A. niger* . This microbiological epidemiology is similar to that of previous reports; however, *Aspergillus* colonisation was excluded from the CPA group in our study [22]. Among the 24 *Aspergillus* strains isolated in fungal cultures, the uncommon species *A. terreus* was isolated from three patients. Notably, two of these patients exhibited *A. fumigatus* coinfection. Treatment of patients with NTM‐PD coinfected with CPA is challenging owing to drug–drug interactions [10, 22, 23]. In our study, treatment had to be changed in five patients with NTM‐PD coinfected with CPA during the observation period. Physicians should carefully consider the risk of CPA coinfection in patients with NTM‐PD.

Previous studies have reported the following risk factors for CPA coinfection in patients with NTM‐PD: fungal balls and cavities with adjacent extrapleural fat, systemic steroid use, cavity formation, emphysema, hypoalbuminemia, older age, male sex, COPD and *M. abscessus* complex [7, 8, 9, 11, 17]. In our study, we demonstrated that male sex, COPD, oral corticosteroid use, and cavity formation are risk factors for CPA complications. These factors may help clinicians identify patients with NTM‐PD who are at a high risk of developing CPA, thereby leading to early CPA diagnosis.

Previous reports have not adequately evaluated patient backgrounds to determine whether CPA complications are independent of poor prognostic factors or whether mortality is high in populations at a high risk of CPA complications owing to case number limitations. We confirmed that CPA comorbidity was associated with a poor long‐term prognosis in patients with NTM‐PD, even after adjusting for confounding factors using propensity score matching. This result corroborates the findings of our previous analysis using the Japanese claims database, which examined cases requiring treatment for both NTM‐PD and CPA coinfection [21]. A recent systematic review of CPA prognosis showed that NTM‐PD as an underlying disease is not an independent risk factor for mortality in patients with CPA [24]. In contrast, our study suggests that in patients with NTM‐PD, the complication of CPA affects prognosis. This highlights the importance of vigilant monitoring of CPA in patients with NTM‐PD, particularly in those with identified risk factors.

The strengths of this study include its large sample size, multicentre design, and long‐term follow‐up. However, certain limitations of this study should be acknowledged. First, this study was retrospective, and unlike prospective studies, selection bias, data quality and completeness, and confounding variables should be considered as potential limitations. Second, there is the possibility of variability in CPA diagnosis. CPA diagnosis is sometimes challenging, and a multicentre study design using medical records may exhibit considerable variation. Surgical resection for CPA was performed in only four patients, and some patients with NTM‐PD without CPA may have remained undiagnosed despite the presence of the fungus ball. *Aspergillus* antibody test can also cause limitations to CPA diagnosis. At the time of the study, *Aspergillus* IgG antibodies were not commercially available in Japan, and *Aspergillus* precipitating antibodies were widely used. A previous study reported that precipitating antibodies have lower sensitivity than IgG antibodies [19]. Third, generalisability outside Japan is limited because the aetiology of NTM differs between Japan and other areas [25, 26]. However, *M. avium* complex was the predominant organism in this study. In many other areas, *M. avium* complex is one of the primary NTM‐PD causative species, albeit with regional variations. Fourth, our study included 45 patients with CPA. However, no subtype analysis of CPA was performed. Systematic reviews and meta‐analyses of CPA have demonstrated that CPA subtypes are associated with mortality and that patients with CCPA, CFPA and SAIA have worse prognoses than those with simple aspergilloma [24]. In our study, 42 patients were diagnosed with CCPA, CFPA or SAIA. Consequently, the impact on the outcome was considered to be minimal.

In conclusion, the findings of this study suggest that the CPA coinfection rate in patients with NTM‐PD is 3.5%. Furthermore, male sex, COPD, oral corticosteroid use, and cavity formation are risk factors for CPA coinfection in patients with NTM‐PD. Additionally, CPA is an independent poor prognostic factor. Clinicians should consider CPA when treating patients for NTM‐PD, particularly those with risk factors. Additionally, in patients with CPA, evaluation for mycobacterial infection, including NTM and *Mycobacterium tuberculosis* , is crucial in cases of clinical deterioration. Extended observational studies of prospective trials are necessary to further elucidate the risk factors and long‐term prognosis of patients with NTM‐PD and CPA coinfection.

## Author Contributions

**Yasuhiro Tanaka:** formal analysis, investigation, writing – original draft. **Shotaro Ide:** investigation, formal analysis, conceptualization, data curation, visualization, methodology, project administration, writing – review and editing, funding acquisition, writing – original draft. **Takahiro Takazono:** conceptualization, methodology, writing – review and editing, project administration. **Kazuaki Takeda:** formal analysis, investigation, methodology, writing – review and editing, resources. **Naoki Iwanaga:** investigation, writing – review and editing. **Masataka Yoshida:** investigation, writing – review and editing. **Naoki Hosogaya:** data curation, resources, writing – review and editing. **Yusei Tsukamoto:** writing – review and editing, investigation. **Satoshi Irifune:** investigation, writing – review and editing. **Takayuki Suyama:** investigation, writing – review and editing. **Tomo Mihara:** investigation, writing – review and editing. **Akira Kondo:** investigation, writing – review and editing. **Tsutomu Kobayashi:** investigation, writing – review and editing. **Yuichi Fukuda:** investigation, writing – review and editing. **Eisuke Sasaki:** investigation, writing – review and editing. **Toyomitsu Sawai:** investigation, writing – review and editing. **Yasuhito Higashiyama:** investigation, writing – review and editing. **Kohji Hashiguchi:** investigation, writing – review and editing. **Minako Hanaka:** investigation, writing – review and editing. **Toshihiko Ii:** investigation, writing – review and editing. **Kiyoyasu Fukushima:** investigation, writing – review and editing. **Kosaku Komiya:** investigation, writing – review and editing, funding acquisition. **Taiga Miyazaki:** investigation, writing – review and editing. **Kazuhiro Yatera:** investigation, writing – review and editing. **Koichi Izumikawa:** methodology, writing – review and editing. **Akitsugu Furumoto:** methodology, writing – review and editing. **Katsunori Yanagihara:** resources, writing – review and editing. **Hiroshi Mukae:** conceptualization, supervision, writing – review and editing.

## Conflicts of Interest

The authors declare no conflicts of interest.

## Supporting information


Data S1.

## Data Availability

The datasets analysed in this study are not publicly available.

## References

[1] J. Adjemian , K. N. Olivier , A. E. Seitz , S. M. Holland , and D. R. Prevots , “Prevalence of Nontuberculous Mycobacterial Lung Disease in U.S. Medicare Beneficiaries,” American Journal of Respiratory and Critical Care Medicine 185 (2012): 881–886. 22312016 10.1164/rccm.201111-2016OC PMC3360574

[2] H. Namkoong , A. Kurashima , K. Morimoto , et al., “Epidemiology of Pulmonary Nontuberculous Mycobacterial Disease, Japan,” Emerging Infectious Diseases 22 (2016): 1116–1117. 27191735 10.3201/eid2206.151086 PMC4880076

[3] V. N. Dahl , M. Molhave , A. Floe , et al., “Global Trends of Pulmonary Infections With Nontuberculous Mycobacteria: A Systematic Review,” International Journal of Infectious Diseases 125 (2022): 120–131. 36244600 10.1016/j.ijid.2022.10.013

[4] K. Harada , H. Hagiya , T. Funahashi , T. Koyama , M. R. Kano , and F. Otsuka , “Trends in the Nontuberculous Mycobacterial Disease Mortality Rate in Japan: A Nationwide Observational Study, 1997–2016,” Clinical Infectious Diseases 73 (2021): e321–e326. 32556251 10.1093/cid/ciaa810

[5] K. Morimoto , N. Hasegawa , K. Izumi , et al., “A Laboratory‐Based Analysis of Nontuberculous Mycobacterial Lung Disease in Japan From 2012 to 2013,” Annals of the American Thoracic Society 14 (2017): 49–56. 27788025 10.1513/AnnalsATS.201607-573OC

[6] C. L. Daley , J. M. Iaccarino , C. Lange , E. Cambau , R. J. Wallace, Jr. , and C. Andrejak , “Treatment of Nontuberculous Mycobacterial Pulmonary Disease: An Official ATS/ERS/ESCMID/IDSA Clinical Practice Guideline,” Clinical Infectious Diseases 71 (2020): e1–e36. 32628747 10.1093/cid/ciaa241 PMC7768748

[7] K. Takeda , Y. Imamura , T. Takazono , et al., “The Risk Factors for Developing of Chronic Pulmonary Aspergillosis in Nontuberculous Mycobacteria Patients and Clinical Characteristics and Outcomes in Chronic Pulmonary Aspergillosis Patients Coinfected With Nontuberculous Mycobacteria,” Medical Mycology 54 (2016): 120–127. 26531100 10.1093/mmy/myv093

[8] K. Furuuchi , A. Ito , T. Hashimoto , S. Kumagai , and T. Ishida , “Risk Stratification for the Development of Chronic Pulmonary Aspergillosis in Patients With *Mycobacterium avium* Complex Lung Disease,” Journal of Infection and Chemotherapy 24 (2018): 654–659. 29705392 10.1016/j.jiac.2018.04.002

[9] K. Fukushima and H. Kida , “New/Different Look at the Presence of Aspergillus in Mycobacterial Pulmonary Diseases. Long‐Term Retrospective Cohort Study,” Microorganisms 9 (2021): 270. 33525485 10.3390/microorganisms9020270 PMC7912930

[10] K. Takeda , T. Takazono , and H. Mukae , “Drug–Drug Interactions in the Management of Non‐Tuberculous Mycobacterial Infections,” Frontiers in Microbiology 15 (2024): 1468383. 39301186 10.3389/fmicb.2024.1468383 PMC11410596

[11] N. Maruguchi , E. Tanaka , N. Okagaki , et al., “Clinical Impact of Chronic Pulmonary Aspergillosis in Patients With Nontuberculous Mycobacterial Pulmonary Disease and Role of Computed Tomography in the Diagnosis,” Internal Medicine 62 (2023): 3291–3298. 36927976 10.2169/internalmedicine.0836-22 PMC10713357

[12] Nontuberculous Mycobacteriosis Control Committee of the Japanese Society for Tuberculosis , Scientific Assembly for Infection and Tuberculosis of the Japanese Respiratory Society , and International Exchanging Committee of the Japanese Society for Tuberculosis , “Guidelines for the Diagnosis of Pulmonary Nontuberculous Mycobacterial Diseases 2008,” Kekkaku 86 (2011): 37–39. 21401004

[13] D. E. Griffith , T. Aksamit , B. A. Brown‐Elliott , et al., “An Official ATS/IDSA Statement: Diagnosis, Treatment, and Prevention of Nontuberculous Mycobacterial Diseases,” American Journal of Respiratory and Critical Care Medicine 175 (2007): 367–416. 17277290 10.1164/rccm.200604-571ST

[14] S. Kohno , K. Tamura , Y. Niki , et al., “Executive Summary of Japanese Domestic Guidelines for Management of Deep‐Seated Mycosis 2014,” Journal of Medical Mycology 57 (2016): E117–E163. 10.3314/mmj.16-00010 27904053

[15] D. W. Denning , J. Cadranel , C. Beigelman‐Aubry , et al., “Chronic Pulmonary Aspergillosis: Rationale and Clinical Guidelines for Diagnosis and Management,” European Respiratory Journal 47 (2016): 45–68. 26699723 10.1183/13993003.00583-2015

[16] Y. Kanda , “Investigation of the Freely Available Easy‐to‐Use Software ‘EZR’ for Medical Statistics,” Bone Marrow Transplantation 48 (2013): 452–458. 23208313 10.1038/bmt.2012.244 PMC3590441

[17] B. W. Jhun , W. J. Jung , N. Y. Hwang , et al., “Risk Factors for the Development of Chronic Pulmonary Aspergillosis in Patients With Nontuberculous Mycobacterial Lung Disease,” PLoS One 12 (2017): e0188716. 29190796 10.1371/journal.pone.0188716 PMC5708732

[18] S. Ishikawa , S. Yano , T. Kadowaki , et al., “Clinical Analysis of Non‐Tuberculous Mycobacteriosis Cases Complicated With Pulmonary Aspergillosis,” Kekkaku 86 (2011): 781–785. 22111386

[19] I. S. Sehgal , K. Soundappan , R. Agarwal , et al., “Prevalence of Chronic Pulmonary Aspergillosis in Patients With Mycobacterial and Non‐Mycobacterial Tuberculosis Infection of the Lung: A Systematic Review and Meta‐Analysis,” Mycoses 68 (2025): e70060. 40265658 10.1111/myc.70060

[20] A. Barac , A. Vujovic , A. Drazic , et al., “Diagnosis of Chronic Pulmonary Aspergillosis: Clinical, Radiological or Laboratory?,” Journal of Fungi 9 (2023): 1084. 37998889 10.3390/jof9111084 PMC10672318

[21] T. Takazono , S. Ide , M. Adomi , et al., “Risk Factors and Prognostic Effects of Aspergillosis as a Complication of Nontuberculous Mycobacterial Pulmonary Disease: A Nested Case–Control Study,” Mycoses 68 (2025): e70022. 39777801 10.1111/myc.70022

[22] M. Fayos , J. T. Silva , F. Lopez‐Medrano , and J. M. Aguado , “Non‐Tuberculous Mycobacteria and Aspergillus Lung co‐Infection: Systematic Review,” Journal of Clinical Medicine 11 (2022): 5619. 36233487 10.3390/jcm11195619 PMC9571699

[23] P. Phoompoung and M. Chayakulkeeree , “Chronic Pulmonary Aspergillosis Following Nontuberculous Mycobacterial Infections: An Emerging Disease,” Journal of Fungi 6 (2020): 346. 33302348 10.3390/jof6040346 PMC7762599

[24] A. Sengupta , A. Ray , A. D. Upadhyay , et al., “Mortality in Chronic Pulmonary Aspergillosis: A Systematic Review and Individual Patient Data Meta‐Analysis,” Lancet Infectious Diseases 25 (2025): 312–324. 39617023 10.1016/S1473-3099(24)00567-X

[25] W. Hoefsloot , J. van Ingen , C. Andrejak , K. Angeby , R. Bauriaud , and P. Bemer , “The Geographic Diversity of Nontuberculous Mycobacteria Isolated From Pulmonary Samples: An NTM‐NET Collaborative Study,” European Respiratory Journal 42 (2013): 1604–1613. 23598956 10.1183/09031936.00149212

[26] S. M. H. Zweijpfenning , J. V. Ingen , and W. Hoefsloot , “Geographic Distribution of Nontuberculous Mycobacteria Isolated From Clinical Specimens: A Systematic Review,” Seminars in Respiratory and Critical Care Medicine 39 (2018): 336–342. 30071548 10.1055/s-0038-1660864

